# “Ancestral recipes”: a mixed-methods analysis of MyPlate-based recipe dissemination for Latinos in rural communities

**DOI:** 10.1186/s12889-022-14804-3

**Published:** 2023-02-01

**Authors:** Ann Marie Cheney, William J. McCarthy, María Pozar, Christina Reaves, Gabriela Ortiz, Diana Lopez, Perla A. Saldivar, Lillian Gelberg

**Affiliations:** 1 Department of Social Medicine Population and Public Health, University of California Riverside School of Medicine, 900 University Ave, Riverside, CA 92521 USA; 2 Conchita Servicios de la Comunidad, Madrid, Spain; 3 University of California Los Angeles Fielding School of Public Health, California, Los Angeles USA; 4 House Institute Foundation, California, Los Angeles USA; 5 University of California Riverside College of Arts, Humanities, and Social Sciences, Riverside, California USA; 6 University of California Los Angeles David Geffen School of Medicine, California, Los Angeles USA

**Keywords:** Diabetes, Chronic disease, Low-income patients, Latinx / indigenous Latin Americans, Purépecha community, MyPlate, Nutrition education, Community-based interventions

## Abstract

**Background:**

The Latinx population experiences some of the highest rates of chronic disease, including obesity and type II diabetes. Such conditions may be especially burdensome in rural Latinx communities that often face barriers to accessing disease prevention resources and public health programs.

**Methods:**

Diverse stakeholders (i.e., patients, community members, system of healthcare clinics, community food bank) tailored an existing cookbook, based on the U.S. Department of Agriculture MyPlate healthy eating and dietary guidelines, for local ingredients, health literacy, and language for rural Latinx and Indigenous Latin Americans. The cookbook recipes were disseminated widely via virtual cooking demonstrations, food distribution events, and social media. Pre- and posttest surveys were used to assess changes in diabetes knowledge measured by the 24-item American Diabetes Association Diabetic Knowledge Questionnaire and confidence in dietary behavior change over time measured by 4 questions of the 17-item Mediterranean Diet Index. A mixed effects, repeated measures analysis was conducted with gender ID, age range and educational attainment included as covariates and assessment interval as the predictor (pretest vs posttest) and change in confidence about adhering to four specific components of the Mediterranean diet. Focus groups elicited information on participants’ motivation and ability to use the recipes and eat healthy foods following the virtual cooking demonstration participation.

**Results:**

A total of 20 virtual cooking demonstrations were conducted and 60 participants completed a pretest survey and 54 a posttest survey, a subsample (*n* = 19) participated in one of three focus groups. Most participants were female, identified as Latinx/Hispanic, were between the ages of 40-49, and spoke Spanish. 17% identified as Indigenous Latin American specifically as Purépecha, an indigenous group from Michoacán, Mexico. Survey and focus group findings indicated at posttest an increase in diabetes knowledge among participants with no prior diagnosis of chronic health conditions and more confidence in limiting sugary beverages and refined wheat pasta/white rice among indigenous participants. Focus group discussions explicated the quantitative findings.

**Conclusion:**

This study brought together patients and key stakeholders committed to addressing the social determinants of health and it mobilized the community to develop culturally vetted health education materials. The findings indicate the need for increased access to evidence-based nutrition education and to culturally appropriate food products that can be easily incorporated into daily food preparation.

## Introduction

The Latinx population, like other major racial-ethnic minority groups in the United States (US) including Pacific Islander and Native American groups, has among the highest rates of obesity, placing this population at significant risk for type II diabetes and cardiovascular disease [1, 2]. According to the Center for Disease Control and Prevention (CDC), the Latinx adult population in the US has a 50% chance of being diagnosed with diabetes at some point in their lifetime [3]. Compared to the US adult population as a whole, Latinos/Hispanics tend to develop diabetes at a younger age and experience greater morbidity (e.g., higher rates of kidney failure, vision loss). Access to clinical care, public health nutrition education, healthy eating, and physical activity is critical to diabetes prevention and management [4]. Following recommended dietary practices such as the Dietary Approaches to Stop Hypertension or DASH diet, which promotes increases in daily fruit and vegetable intake, result in better quality diets and reduces chronic disease risk [5].

Despite significant health needs, the Latinx patient population faces challenges to engagement in disease care management and prevention. This is particularly pronounced for the Latinx population in rural communities, which often struggles to access basic resources including healthy foods and the nutrition knowledge needed to prevent obesity and related conditions such as type II diabetes [6, 7]. Not only does this patient population have limited access to these resources, but they are also vulnerable to inequalities in health due to their citizenship status, ethnicity, indigeneity status, and rural geographic location [8].

The health of the Latinx population in rural America is a pressing public health concern. Over the past decade, the Latinx population in rural America has increased dramatically from a population of 2 million to 6 million [9]. The majority, concentrated in border states, work in low-wage jobs (e.g., agribusiness, construction, or the service industry). It is well-known that people in rural communities face significant barriers to accessing healthcare services and public health programs. Rural areas often lack primary and specialty healthcare providers, hospitals and emergency care services, and public health programs for health promotion and disease prevention [10, 11]. Thus, approaches to reduce access barriers for healthcare and public health services among rural Latinx populations are desperately needed.

**Fig. 1:**
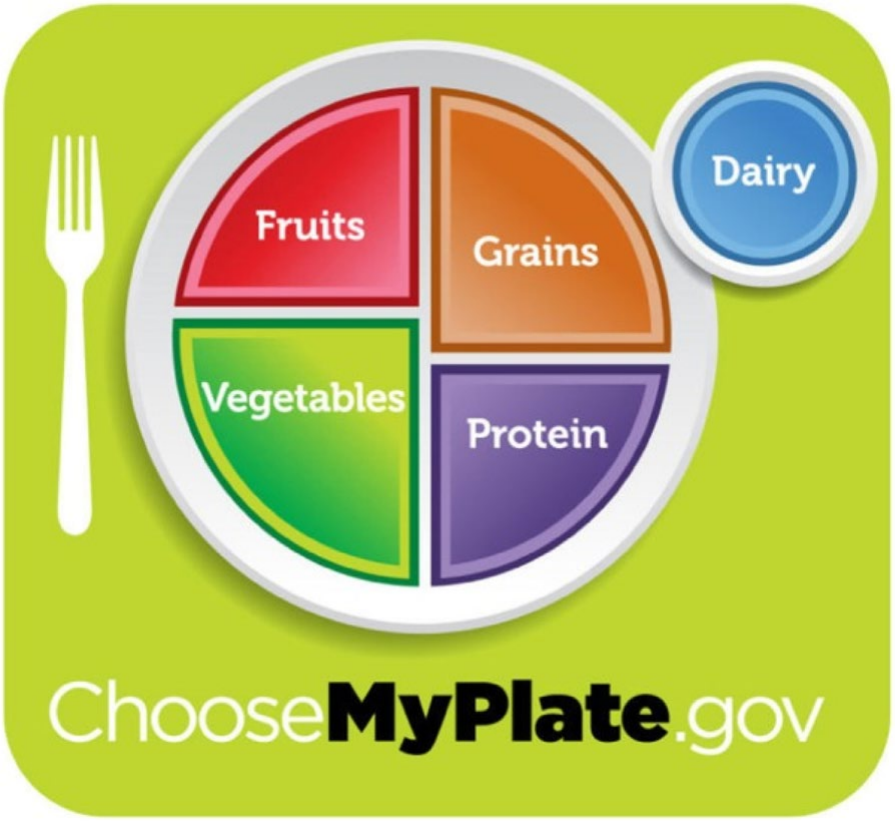
U.S. Department of Agriculture MyPlate Icon for Recommended Dietary Guidelines, www.choosemyplate.gov

Nutrition education through cooking programs is increasingly utilized as part of public health programs for health promotion and disease prevention among low-income communities [12–14]. In line with ongoing public health efforts, this study brought together patients, a healthcare system, and a community food bank to develop patient-centered and community-informed dissemination strategies focused on increasing access to healthy foods and nutrition education. MyPlate.gov is the US Department of Agriculture’s website explicating federal nutrition guidelines. The MyPlate icon depicts five food groups: fruits, vegetables, grains, protein, and dairy that vary in size and color, and is a visual aid used to promote food variety and translate dietary guidelines into practice (see Fig. 1). The icon is simple and easy to interpret. There are four food groups on the plate with dairy on the side. Fruits and vegetables occupy more space on the plate while grains and protein occupy the least amount of space on the plate. Previous research found that MyPlate-based-recipes and interventions reduced excess body fat and improved mental health and quality of life in low-income, urban Latinx patients [15]. Our study focused on the dissemination of MyPlate recipes to low-income, rural Latinx communities and patients to support diabetes prevention and management.

### MyPlate-based recipes

This project builds on the findings of a 12-month comparative effectiveness research (CER) trial that compared a community health worker-delivered calorie counting/portion control approach to the MyPlate high-satiety/high-satiation foods and no calorie-counting approach involving primarily urban Latinx patients who were overweight or obese and accessed services at a federally qualified healthcare center (FQHC) in southern California [15]. At the 12-month follow-up assessment, participants in both intervention arms had less abdominal fat, felt fuller and were more satisfied with meals, had improved mental health and quality of life, and were highly satisfied with their assigned weight loss program [16, 17]. Participants in the MyPlate arm had lower blood pressure 6 months after starting the study but not at the 12-month follow-up. The qualitative assessment of the MyPlate intervention found this approach to be a particularly appropriate vehicle for communicating nutritional priorities to the mostly low-income, Latinx patient population because it required minimal literacy and numeracy skills and included cooking classes.

MyPlate’s emphasis on fruits, vegetables, legumes, and nuts is well-suited to immigrants raised on a traditional Mesoamerican diet of maize, beans (e. g., black, pinto), and squash (e.g., pumpkin, acorn squash) [18]. Furthermore, as this study and others have found, having bilingual, bicultural community health workers (i.e., promotores de salud) as change agents, rather than masters-level health educators, is consistent with community health practices in low-income Latinx communities [19]. The use of MyPlate guidelines for behavior change practices of weight control is facilitated by the increased satiety, emotional wellbeing, and quality of life associated with consuming more minimally processed foods [20–22].

Such work supports the need for culturally vetted health education to help patients understand how to prevent and manage chronic conditions via changes in nutrition and physical activity. It also supports the need to increase both access to evidence-based nutrition knowledge and the ability to incorporate that knowledge into daily food preparation.

## Methods

### Project overview

This study was carried out from January 2020 to October 2021. It builds on previous research on community health priorities among Latinx immigrants in inland Southern California’s Eastern Coachella Valley (ECV), in which diabetes and obesity were community health concerns. Furthermore, while community members indicated access to food items, including fresh fruits and vegetables, some items would be discarded as they were unaware of how to incorporate them into daily cooking [6]. With knowledge about our research in the ECV and research in the Los Angeles area on chronic disease prevention among Latinx urban communities [15], our clinical partner, Borrego Health, facilitated bringing these two investigative teams together.

Community-based participatory research (CBPR), a collaborative approach to form equitable partnerships and share power and decision-making with patients and key stakeholders, was employed in all phases of the project [23]. In line with this approach, we convened a steering council that met quarterly to offer input and direction and ensure that project goals were met. This project included three phases: 1) tailoring the existing MyPlate recipes to rural low-income Latinx primary care patients, 2) disseminating the tailored recipes to this community, and, 3) evaluating the impacts of the tailored recipes on nutrition knowledge and confidence to undertake desired behavior changes.

A key part of this project was the use of the Analysis, Design, Develop, Implement, and Evaluate (ADDIE) model which informed each step of the tailoring of the recipes and cookbook. ADDIE is an instructional design methodology that provides a framework to incorporate learner perspectives into program design and development [24]. The steering council identified eight patients and six stakeholders who met seven times for 2 hours each to tailor the recipes and cookbook. During workgroup meetings, we found the community was most concerned about diabetes and its precursor metabolic syndrome symptoms (especially central obesity, high blood sugar, and high systolic blood pressure). We therefore tailored the recipes and cookbook to address the needs of prediabetic and diabetic patients. This involved interspersing diabetes education throughout the cookbook and having recipes reviewed by a physician, an academic with expertise in diet, and a nutrition educator specializing in treating patients with diabetes.

**Fig. 2:**
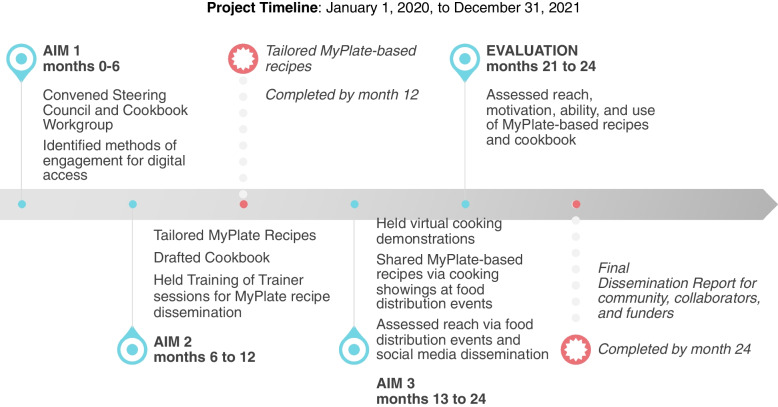
Project activity over two-year award period

Figure 2 provides an overview of each phase of the project by aim during the two-year award period. The University of California Riverside and University of California Los Angeles Institutional Review Boards (IRB) approved all data collection procedures, study instruments, and consent documents. Electronic consent was obtained from all participants prior to data collection. For participants who opted into the focus groups, an information sheet was shared with them and a second verbal consent was obtained prior to the start of all focus groups.

### Setting and partnerships

This study was conducted in partnership with a system of FQHCs in inland southern California and FIND Food Bank, a member of Feeding America. It was carried out in the ECV, located in the 45-mile-long Coachella Valley. The rural unincorporated areas of the eastern valley, Mecca, North Shore, Oasis, and Thermal are among the richest agricultural areas in the world, contributing as much as $600 million in annual agricultural production [25]. This rural region is also one of the most impoverished areas of California and is well-known for its underinsured Latinx immigrant population who live below the poverty line in substandard conditions [26, 27].

The system of  FQHCs provides primary and specialty care to low-income, racial-ethnic minority patients in the ECV. There are eight clinics in the Coachella Valley region, two of which are rural clinics that serve Latinx farm working communities including an Indigenous, predominantly Purépecha-speaking, Mexican patient population. Of those accessing medical care within the two rural clinics, patient data collected on unique visits from July 2017 to 2019 indicated over a third of adult patients suffer from uncontrolled diabetes (HbA1c > 9). The healthcare system has several services for patients living with diabetes or pre-diabetes, including referrals to health educators who provide nutrition education and monitor eating and physical activity as well as referrals to CalFresh, California’s version of the federal Supplemental Nutrition Assistance Program (SNAP) for low-income people in need of healthy and nutritious foods (https://www.cdss.ca.gov/calfresh). Social care for diabetes, however, extends beyond what can be done in the clinic to address the social determinants of health that impede diabetes management and prevention. For this low-income, Latinx immigrant and Indigenous Mexican patient population, that includes access to healthy food.

Despite being the backbone of the American Food System, this rural Latinx population cannot easily access the fresh fruits and vegetables they harvest [28]. Everyday consumption of fresh fruits and vegetables is needed to help manage chronic illnesses such as obesity and type II diabetes. Most of the food harvested in the region is shipped outside of the ECV to wealthier markets, making it difficult for food insecure individuals to access or afford what they harvest and prompting many to access available produce at free community food distributions.

FIND Food Bank is the Coachella Valley’s regional food bank and hub for information and action on hunger and food insecurity—this organization provides food assistance to food insecure individuals and families in the ECV. FIND Food Bank has five agency sites (i.e., partnering agencies where food is distributed) and nine mobile pantries in the eastern valley. Through these sites, they provide food assistance to approximately 11,130 individuals and 3180 families per month. Importantly, FIND food distribution follows the USDA MyPlate healthy eating recommendations.

### Phase 1- tailoring MyPlate recipes

Over a series of seven, 90-minute virtual workgroup sessions, 6 community members, including patients living with diabetes, two FIND Food Bank outreach workers, a public health researcher with expertise in nutrition, a primary care physician researcher, and two medical students (*n* = 12) completed the first three steps of the ADDIE model: analysis, design, and development. They reviewed the original 28 recipes utilized as part of the MyPlate high-satiety/high-satiation intervention described above and considered their appropriateness for Latinx patients in the ECV. The workgroup was charged with tailoring the MyPlate recipes to account for local food resources, cultural acceptability, health literacy levels, and language. Additionally, they were asked to consider whether recipes were appropriate for patients living with diabetes. During cookbook workgroup meeting discussions, patients and community members discussed the importance of providing recipes and nutrition education appropriate for mothers with gestational diabetes as well as persons preparing meals for prediabetic and diabetic family members.

Several recipes were omitted from the original cookbook, either because the plate/meal was not commonly prepared by members of the community (e.g., pizza, which requires an oven to bake) or contained too many carbohydrates (e.g., torta, which contains beans and bread). During workgroup meetings, participants were introduced to the concept of the ‘three sisters’—corn, bean, and squash, a Mesoamerican, pre-Columbian food tradition that their grandparents most likely farmed and ate, in which the planting of the ‘sisters’ together kept the soil fertile and eating them together yielded all the essential amino acids [29, 30]. Workgroup members were asked to consider ways to incorporate Mesoamerican food traditions into the cookbook as well as nutrition education addressing community needs and food and eating practices. The final cookbook included 45 recipes, a description of the ‘three sisters,’ and 12 illustrations with short educational nutrition dialogues.

Throughout the workgroup meetings, community members identified additional recipes, such as traditional dishes unique to their home community (e.g., Atapakua from Michoacán, Mexico). The inclusion of such dishes conveyed important nutrition education and information emphasizing the importance of connecting to past food traditions for healthy living and longevity. Melodramatic dialogues, like the use of telenovelas in public health messaging, were interspersed throughout the cookbook to convey nutrition education meaningful to the community. This included the importance of eating grains and fruit for breakfast rather than coffee and pastries (the typical breakfast food in modern day Mexico); the importance of eating healthy snacks between meals to reduce overeating calories, a practice uncommon in many Mexican food traditions; and the use of a fist to measure one cup of food. Throughout the process, a community-based healthcare provider treating patients in two rural clinics in the ECV reviewed the recipes and dialogues and offered insight based on experience providing nutrition and diabetes education to Latinx patients in the region.

With input from steering council and cookbook workgroup members, we titled the cookbook: “Ancestral Recipes: From My Grandma’s Kitchen to Yours,” which was first written in Spanish and then translated into English and Purépecha [31]. We reviewed the final recipes in the cookbook and selected three for production of short (2 to 5 minutes) video cooking demonstrations produced in English, Spanish, and Purépecha. These videos were uploaded to the partnering site’s YouTube channel, as well as posted on the project’s Facebook page. Additionally, we selected 22 of the 45 recipes to be passed out as single recipe cards during community food distribution events held by FIND Food Bank and 20 of the 45 recipes to be featured during the Facebook Live cooking demonstrations posted on the project’s Facebook page. Each virtual cooking demonstration featured one or two recipes (see Table 1 for featured recipes by dissemination strategy).

**Table 1:** MyPlate-based Recipe Dissemination

Dissemination Strategy	Description	Recipes disseminated	Number of events/videos	Direct reach
Virtual Cooking Demonstrations	From February to July 2021, we hosted virtual cooking demonstrations on Zoom, simultaneously streamed on Facebook Live.	Atapacua* Chicken and vegetable soup Chicken meatballs Chicken salad Chicken tinga Corn and green chile salad Crazy cucumbers Fish soup Fish wrapped in corn husks Gallina pinta** Guacamole Homemade salsa Huevos rancheros Lentil soup Mexican squash Mole verde Nopal salad Pea soup Pico de gallo Quesadilla Spinach tortillas Stuffed vegetables Tuna salad Vegetable ceviche	20 videos	132 live attendees
Cookbook dissemination	We distributed physical copies of the cookbook to audience members in our Facebook Live Cooking Demonstrations.	All 45 recipes in the cookbook	20 events	72 community members
Professional Video		Fish Wrapped in Corn Husks Mexican Squash Oatmeal	9 videos	*Not assessed*
Cooking Showings	Recipe handout at food distribution sites and accompanying ingredients to prepare the recipe. Audio of the Facebook Live cooking demonstration could be tuned into via radio while waiting for food.	Atapacua Ceviche with vegetables Chicken and vegetable soup Chicken meatballs Chicken tinga Chili with sweet potatoes Corn and green chile salad Fish soup Fish wrapped in corn husks Gallina pinta Guacamole Huevos rancheros Lentil soup Mexican squash Mole verde Nopal salad Quesadilla Spinach tortillas Split pea soup Stuffed vegetables Tuna salad	25 events	8497 households
Recipe distribution	Three trained community health workers/*promotores* distributed recipe cards during food distribution events and shared the QR code to download the cookbook as well as access the videos of the virtual Facebook Live cooking demonstrations.	Atapakua Ceviche with vegetables Chicken and vegetable soup Chicken meatballs Chicken tinga Chili with sweet potatoes Corn and green chile salad Fish soup Fish wrapped in corn husks Gallina pinta** Guacamole Huevos rancheros Lentil soup Mexican squash Mole verde Nopal salad Quesadilla Spinach tortillas Split pea soup Stuffed vegetables Tuna salad	64 events	23,591 households

*Atupacua is a typical dish from Purépecha communities in Michoacán, Mexico that combines squash flowers, Mexican squash, corn, and tomatoes with garlic, onion, and spices

**Gallina pinta is a typical dish from the state of Sonora, Mexico that combines beans, hominy corn, and a beef bone for flavoring

### Phase 2- disseminating tailored MyPlate recipes

The fourth step of the ADDIE model, implementation/dissemination, was carried out from Spring to Fall 2021. We used McCormack et al.’s [32] recommendations for successful dissemination strategies, which include a focus on reach of evidence, motivation to apply evidence, and ability to use and apply evidence. Dissemination activities included virtual cooking demonstrations, cooking videos, and recipe cards.

Prior to dissemination of the cookbook and recipes, team members held a six-hour training for 10 individuals, including community health workers (promotores de salud), FIND Food Bank outreach workers, and health educators in the partnering health system. We used the Center for Disease Control’s Training of Trainers (ToT) model [33] as a method to prepare trainees to hold virtual cooking demonstrations and share nutrition education with patients and stakeholders, as well as become the “experts” and train new, less experienced trainees and prepare them to present information, respond to trainee questions and concerns, and lead activities. This model builds community capacity for health promotion [34].

Of the trainees who participated in the training, six participated in one or more of the cooking demonstrations either as the chef or health educator. Trainees then recruited another five community members, who were trained to hold a cooking demonstration during which they discussed the cookbook recipes and provided health education; these trainees held at least one cooking demonstration. Trainees disseminated recipes and nutrition education through virtual cooking demonstrations from their home kitchens via Zoom, or live streamed on Facebook Live. Attendees of the virtual cooking demonstrations were mailed a hardcopy of the cookbook. Trainees also disseminated recipe cards and boxes with some of the ingredients needed to cook the recipe during food distribution events in the ECV. The partnering organizations created a page for the cookbooks on their websites and promoted the virtual cooking demonstrations on social media prior to the event. The system of health centers sent out text messages to Spanish-speaking patients who accessed services in the ECV informing them about the cookbook.

### Phase 3- evaluating MyPlate recipes on nutrition knowledge and behavior change

Our evaluation, the final step of the ADDIE model, assessed the reach of MyPlate recipes among the targeted patient and community groups, the increase in diabetes knowledge and dietary behavior change, and community members’ ability and motivation to use the tailored MyPlate recipes. Social media metrics (views, shares, comments) were used to assess reach and audience demographics (gender, age, geographic region). Pretest and posttest surveys assessed nutrition knowledge and dietary behavior change. We anticipated that among the total posttest sample, the 10 participants identifying themselves as indigenous would be more confident of being able to adhere to Mediterranean diet components and relatedly, would be less likely to be classified as obese compared to the 44 respondents identifying themselves as Latinx. Focus groups elicited information on ability and motivation to use the recipes, as well as behavior change post participation in the cooking demonstrations.

#### Pre- and posttest surveys data collection

We recruited participants from the system of FQHCs and networks of the original trainees. A health educator within the system of FQHCs identified patients who had been diagnosed with pre-diabetes or diabetes and invited them to participate in a cooking demonstration and surveys. An original trainee recruited participants from their social and family networks to participate in the research. Eligible participants were 18 years or older, lived in the ECV, and had access to Zoom, Facebook Live, or WhatsApp to watch the cooking demonstration. Interested patients shared their contact information with the study team, who invited them to participate in the research. Participants completed the 24-item American Diabetes Association Diabetes Knowledge Questionnaire [35] and 4 questions from 17-item Mediterranean Diet (MedDiet) Index [36] at pre- and posttest.

#### Health status

Participant body mass index (BMI) was calculated from self-reported height and weight; obesity was defined as a BMI of 30 or higher. Questions from the CDC’s National Nutrition and Health Examination Survey (NHANES) were used to assess past history of chronic health diagnoses for diabetes (e.g., “has a medical doctor ever diagnosed you with diabetes?”) as well as high blood pressure (“has a doctor or other health professional ever told you that you have. ..? ”) [37]. Current physical activity was assessed by the total number of days 10 minutes or more of vigorous physical activity (heavy lifting, digging, aerobics, fast bicycling) was done in the last week.

#### Diabetes knowledge

All 24 items from the Diabetes Knowledge Questionnaire, a reliable and valid measure of diabetes-related knowledge that can be administered in English and Spanish, were used to assess diabetes knowledge [35]. The total number of correct answers was calculated for each respondent, which constituted the respondent’s diabetes knowledge score.

#### Dietary behavior change

Four items from the 17-item Mediterranean Diet (MedDiet) Index were used to assess patients’ dietary habits; specifically, their consumption of foods known to contribute to diabetes and high cholesterol, including sweetened sugary beverages and red meat [38, 39]. We also sought to assess patients’ knowledge of the health benefits of whole grains versus processed grains. We therefore included the following four items from this scale: 1) “consume whole grain cereals and pasta ≥5 times per week,” 2) consume ≤1 serving (1 serving = 100-150 g) of red meat, hamburgers, or meat products (ham, sausage, etc.) per week, 3) “do not add sugar to beverages (coffee, tea); instead, replace sugar with non-caloric artificial sweeteners, and 4) reduce consumption of pasta or rice < 3 servings per week (unless the pasta or rice are whole grain products). Participants were asked to rate, on a scale of 1 to 3 (1 = Cannot do it; 2 = I can do it; 3 = I am sure I can do it) their confidence in their ability to consume the foods on the following list or to consume them in the quantity suggested.

#### Sociodemographics

Participant characteristics were collected at pretest, including age, gender, racial/ethnic heritage, country of origin, maternal language, health insurance coverage, marital status, educational attainment, and employment status, including employment as farm laborer.

#### Pre- and posttest surveys data analysis

For descriptive analyses, comparisons of the baseline distribution versus the follow-up distribution were evaluated using chi square tests. For cross-sectional analyses of the questionnaire data, continuous outcomes such as BMI were evaluated using regression; binary outcomes were evaluated using logistic regression and categorical outcomes were assessed using ordered logit. Mixed effects regression modeling was used to evaluate change in knowledge between baseline and follow-up. This type of regression yields robust estimates in the presence of missing data. Gender, age and educational attainment were included as covariates in all regression analyses. The Benjamini-Hochberg procedure was used to correct for inflation in type I error caused by the testing of multiple, related hypotheses [40].

Objectively assessed weight and height showed that 85% of U.S. adults diagnosed with diabetes were either overweight or obese compared to 67% without diabetes [41]. To check whether the self-reported weight and height reported by respondents in this survey were accurate, BMI was regressed onto a dummy variable for diabetes diagnosis and gender, age and educational attainment were included as covariates [42]. Stata 13 was used for all analyses.

#### Cooking demonstrations

Satisfaction with the cooking demonstrations was collected at posttest, which assessed perceptions of the amount of information shared, usefulness of cooking demonstrations to encourage consumption of vegetables and plant-based foods, and the usefulness of recipes. Participants were also asked the likelihood of recommending the program (cookbook, recipes, cooking demonstrations) to family members and friends.

#### Focus groups

As indicated by Guest et al. [43], for non-probability samples 80% of themes can be identified with two to three focus groups. A total of three focus groups were conducted (a subsample (*n* = 19) in Spanish) to identify themes directly related to ability and motivation to use the tailored MyPlate recipes. Study team members with expertise in focus group facilitation conducted these groups using a semi-structured interview guide to elicit responses on the following topics: perceptions of the cooking demonstrations, the cookbook and its recipes and nutrition education dialogues, recipe ingredients (affordability and accessibility), and ability and motivation to incorporate the recipes in daily cooking. Focus groups were audio recorded and transcribed using Word dictation software. All transcripts were checked for accuracy—team members listened to the audio recording while simultaneously reviewing the transcribed text and editing for accuracy. A rapid qualitative analytic approach involving the creation of a structured template aligning with the interview guide questions was used to create summaries of each focus group, as well as to identify themes and exemplar quotes [44]. Narrative text was then used to converge or validate the conclusions reached, based on the quantitative analysis of pretest and posttest survey data [45].

## Results

### Overview- dissemination efforts

Table 1 includes an overview of the dissemination strategies, brief description of each strategy, the total number of events or videos, and direct reach. Between February and July 2021, we hosted a total of 20 cooking demonstrations on Zoom, simultaneously streamed on Facebook Live, and we distributed 72 physical copies of the cookbooks to audience members in the ECV. This was followed by 25 cooking demo showings, in which FIND Food Bank provided tailored MyPlate recipe cards and accompanying ingredients to 8497 households serving approximately 34,577 people across the food distribution sites. During these events, FIND Food Bank played the audio of the cooking demonstration of the recipe in distribution via the radio. Community members were encouraged to turn on their car radio and listen to the demonstration while waiting in line for their food. Additionally, three trainees trained by the original trainees in the training of trainer session attended 64 food distribution sites handing out recipes to 23,591 households and representing approximately 95,694 people. During these events, trainees shared recipe cards along with a business card with a QR code to download the free electronic version of the cookbook. While clients awaited their food box, FIND Food Bank staff and promotores who had participated in the training and cooking demonstrations referred community members to the electronic version of the cookbook and talked about the nutrition dialogues in the cookbook and basic information about diabetes [46], risk for diabetes [47], and symptoms of diabetes [48].

### Pretest and posttest survey participant characteristics

Sixty participants completed a pretest survey before the virtual cooking demonstrations, of whom 54 of the 60 participants completed a posttest survey after the cooking demonstrations (six participants dropped out) equating to a 93% completion rate. The pretest participants’ ages ranged from 20 to 70+ years old, with one-third of participants (32.8%) being between the ages of 40 to 49 years. Out of the total 60 respondents, 87% identified as female, 10% identified as male, and 3% provided no response. Most participants (82.8%) identified as Latinx/Hispanic. The remaining 17.2% identified as Indigenous people formerly residing in Latin American countries.

44.8% had only completed elementary school and 43.1% were currently unemployed. Nearly half (46.7%) had been diagnosed with diabetes, 31.7% with hypertension and 50.9% had a BMI of 30 or above. We found that indigenous participants were significantly less likely to be obese compared to Latinx/Hispanic participants (aOR = 5.44; 95% CI: − 1.34, 22.06; *p* = .018). Table 2 describes the sample characteristics and compares pretest to posttest results on the surveys.

**Table 2:** Characteristics of pretest and posttest survey participants, among low-income adult patients with diabetes or prediabetes living in the Eastern Coachella Valley, California, and participating in the MyPlate demonstration project

Variable	Pretest		Posttest		*P*-value^‡^
	N*	%	N	%	
Sample size	60		54		
Gender identity					
Male	6	10.3%	5	9.3%	0.847
Female	52	89.7%	49	90.7%	
Age (years)					
20-29	5	8.6%	5	9.3%	0.999
30-39	10	17.2%	10	18.5%	
40-49	19	32.8%	16	29.6%	
50-59	14	24.1%	14	25.9%	
60-69	7	12.1%	6	11.1%	
70+	3	5.2%	3	5.6%	
Education					0.986
0 to 6 years	26	44.8%	26	48.2%	
7-12 years	11	19.0%	10	18.5%	
HS degree/GDE	12	20.7%	10	18.5%	
13+ years	9	15.5%	8	14.8%	
Marital status					
Single	3	5.2%	3	5.6%	0.992
Widowed	5	8.6%	5	9.3%	
Divorced	4	6.9%	3	5.6%	
Married	46	79.3%	43	79.6%	
Ethnic heritage					
Indigenous	10	17.2%	10	18.5%	0.860
Hispanic/Latino	48	82.8%	44	81.5%	
Ever diagnosed with diabetes					
No	32	53.3%	29	53.7%	0.968
Yes	28	46.7%	25	46.3%	
Ever diagnosed with hypertension					
No	41	68.3%	36	66.7%	0.849
Yes	19	31.7%	18	33.3%	
BMI classification					
< 25	8	14.0%	8	15.7%	0.991
25 to < 30	20	35.1%	17	33.3%	
30 to < 40	24	42.1%	21	41.2%	
40+	5	8.8%	5	9.8%	
Employment status					
Unemployed	25	43.1%	25	46.3%	0.987
Part-time	18	31.0%	16	29.6%	
Full time	5	8.6%	4	7.4%	
Other	10	17.1%	9	16.7%	
Are you a farm worker?					
No	34	58.6%	31	57.4%	0.897
Yes	24	41.4%	23	42.6%	
Maternal language					
English	3	5.4%	2	3.8%	0.984
Purépecha	5	9.1%	5	9.6%	
Spanish	45	81.8%	43	82.7%	
Zapotec	2	3.6%	2	3.8%	
Where born?					
Latin America, including Mexico	54	93.1%	51	94.4%	0.770
Born in the U.S.	4	6.9%	3	5.6%	
Do you have health insurance?					
No	13	22.8%	13	24.5%	0.832
Yes	44	77.2%	40	75.5%	
Weekly physical activity level					
Inactive all days	28	46.7%	25	44.6%	0.971
Active 1-4 days/week	13	21.7%	13	23.2%	
Active 5-7 days/week	19	31.7%	18	32.1%	

*Note. Sample sizes do not always add up to *N* = 60 (baseline) and *N* = 56 (follow-up) because of missing data

‡Note. *P*-value reflects results of chi square test comparing the posttest distribution to the corresponding pretest distribution

### Cooking demonstration participant satisfaction and perspectives

Overall, participants were highly satisfied with the cooking demonstrations and perceived the demonstrations to have motivated them to cook healthier meals. Only a minority (37.5%), however, said that they were satisfied with the amount of information shared with them; 58.9% said that they thought that an excess of information had been provided. By contrast, 98.2% indicated the cooking demonstrations were very useful for encouraging them to eat new vegetables and other plant foods, and 98.2% indicated that the featured recipe was very useful. Similarly, 98.2%indicated they would recommend the cooking demonstrations to their family members and friends.

### Survey results

#### Diabetes knowledge

The brief exposure to a cooking demo/health education did not increase the total diabetes knowledge score. When examined individually, more than half (*n* = 14) of the 24 questions comprising the diabetes knowledge questionnaire elicited 80% or more correct answers at baseline, allowing little improvement from pretest to posttest.

Association of body composition with disease diagnosis. Participant BMI values were significantly or near-significantly associated with self-reported diabetes and hypertension status, even after controlling for gender, age and educational attainment (b_diabetes_ = 3.33; 95% CI: 0.12, 6.55; *p* = .043; b_hypertension_ = 3.20; 95% CI: 0.33, 6.73; *p* = .075).

Self-reported likelihood of adherence to four Mediterranean diet components, by indigeneity status.

Respondents as a whole reported increased confidence after exposure to a cooking demonstration/ health education that they could adhere to two of four components of the Mediterranean diet (b_addedsugar_ = 0.24; 95%CI: 0.02, 0.46; b_redmeat_ = 0.5; 95%CI: 0.02, 0.98) but application of the Benjamini-Hochberg correction for multiplicity of hypothesis-testing reduced this to a finding of increased confidence following exposure to a cooking demo/ health education only for adherence to the Mediterranean diet limit on sugary beverages [40].

Indigenous respondents were consistently more confident than Latinx respondents that they would be able to adhere to three of the four Mediterranean diet components studied (b_addedsugar_ = 0.64; 95%CI: 0.26, 1.02; b_pasta_ = 0.67; 95%CI: 0.29, 1.05; b_whgrain_ = 0.68, 95%CI: 0.29, 1.07). Application of the Benjamini-Hochberg correction did not change these results.

**Fig. 3:**
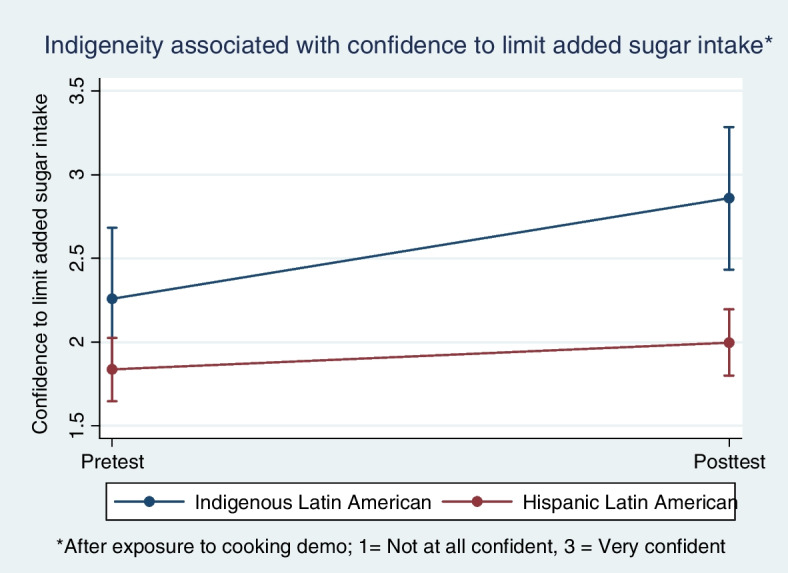
Effect of cooking demonstration on boosting respondent confidence that they could limit weekly intake of adding sugar to beverages (e.g., coffee or tea)

After stratifying by endogeneity status, respondents reported increased confidence that they could adhere to three of four components of the Mediterranean diet (b_redmeat_ = 0.5; 95%CI: 0.02, 0.98; b_addedsugar_ = 0.6; 95%CI: 0.09, 1.11; b_pasta_ = 0.6; 95%CI: 0.08, 1.12). Application of the Benjamini-Hochberg procedure for correcting for the inflation of type I error rendered the increased confidence in one’s ability to limit red meat intake to no more than 150 g per week no longer statistically significant but the increased confidence associated with the component limiting added sugar consumption and the component limiting consumption of refined wheat pasta and white rice were still statistically significant. The general pattern of results across the four measures of adherence to Mediterranean diet components is well-represented by Fig. 3.

As expected, indigenous respondents consistently reported a higher likelihood of adherence to all four Mediterranean diet components than Latinx respondents at posttest (b_whgrain_ = 0.34; 95%CI: 0.08, 0.60; b_addedsugar_ = 0.43; 95%CI: 0.19, 0.67; b_redmeat_ = 0.35; 95% CI: 0.12, 0.59; b_pasta_ = 0.47; 95%CI: 0.23, 0.71) even after applying the Benjamini-Hochberg correction. The pattern of results for adherence to the Mediterranean diet limitation on adding sugar to beverages was similar to the pattern of results observed for the other three Mediterranean diet components tests (see Fig. 3).

Participants previously diagnosed with diabetes did not experience a statistically significant boost in confidence that they could limit intake of red meat/processed meats to 150 g per week (b = .08; 95% CI: − 0.08, 0.23; *p* = .3350) whereas those respondents without diagnosed diabetes did experience a significant boost in confidence from pretest to posttest. (b = 0.16, 95% CI: 0.02, 0.31; *p* = .0266). Similarly for the Mediterranean diet component limiting sugar beverage consumption, respondents without diabetes reported greater confidence following exposure to a cooking demo/health education (b = 0.38; 95%CI: 0.08, 0.68) whereas respondents with diabetes did not. Diabetes status was unrelated to pretest-to-posttest changes in confidence to limit refined carbohydrate consumption and to eat the prescribed number of whole grain servings per week. After applying the Benjamini-Hochberg correction, the change in confidence that the respondent without diabetes would be able to limit their red meat intake to no more than 150 g per week became statistically nonsignificant but the experimental effect on limiting the adding of sugar to beverages by participants without diabetes was preserved.

### Focus group findings

The cooking demonstrations, which included CDC-based diabetes and nutrition education presented by a community member, provided accessible information and ways to incorporate the recipes into daily food practices. Participants indicated that the combination of both diabetes education, and the cooking demonstration helped to establish knowledge around diabetes or broaden it and made them consider the impact of foods components (e.g., salt, fat) on health outcomes. For instance, a participant commented: “I had little knowledge of diabetes, the types of diabetes that exist and the consequences that people can have when consuming too much fat, too much salt.”

#### Ability and motivation to use the recipes

In general, participants indicated an increased abilty and motivation to use the recipes. They especially liked the design and colors of the cookbook and the inclusion of the three sisters. “I really like it [the cookbook], because it has a lot of colors, the presentation is really attractive. It has very colorful pictures, the dolls [three sisters]—I feel that we identify with it [the cookbook].

The recipes were easy to follow, utilized ingredients commonly used by women in the study, and were easily incorporated into daily cooking.

Many of the ingredients are ones that we already use, they are part of our cooking. But it [the cookbook] teaches us how we can combine them [the ingredients] in different ways... and everything is understandable. I feel like it [the cookbook] is really great.

Others commented on specific recipes and the ease with which they could prepare favorite dishes and incorporate them into daily cooking. This participant talked about preparing Gallina Pinta, a soup with beans and hominy corn and flavored with beef. The recipe in the Ancestral Recipes cookbook reduces red meat consumption by using a beef bone for flavoring and recommends incorporating Mexican squash for inclusion of the three sisters (corn, bean, squash). “I love Gallina Pinta. This is a very rich dish and very tasty. But I didn’t know how to make it well. This [recipe] was explained well... in how it’s explained and how it’s prepared.” As partipcants shared, both the recipe as outlined in the cookbook and the virtual Facebook Live cooking demonstration offered guidance on how to prepare dishes.

#### Dietary behavior change

Across the focus groups, participants discussed shifts in meal preparation after having participated in the cooking demonstration and reviewing the recipes. A Latina participant commented: “It is not necessary to make meat for dinner, because sometimes there are recipes, which is what we have at home, we can make them.” Similarly, a woman identifying as Purépecha commented:

I love cooking, the one [recipe], I forget, it is the cactus [ensalada de nopa recipe]. I eat cactus nearly every day, and the zucchini, corn [Mexican squash recipe]. I like them [zucchini] in a soup, or steamed I make it. And well, I love it a lot because as they [doctors] also told me that I have diabetes.

As the above quotes illustrate, the recipes in the cookbook and the cooking demonstrations motivated dietary behavior change. For instance, when asked to comment on the nutrition education provided during the cooking demonstrations, a participant said: “The talks were very interesting. In respect to the recipes, I have realized that everything is going to be a change of our food to eat healthier.” Participants also emphasized the natural elements of the food ingredients in the recipes and their importance to health and wellbeing: “They [ingredients] are from nature that our mother earth gives us, which is what keeps us healthier.”

Focus group participants also discussed the importance of reducing red meat and incorporating more fruits and vegetables into their diets. This is evident in the following comment made by an Indigenous Latina: “I try to avoid these things [red meat], and it’s so important for you to eat fruits and vegetables—and I am liking eating them.” Participants talked about [red] meat as having a negative effect on their bodies and vegetables as contributing to wellbeing:

If we eat too much meat in excess, our bones hurt, or our feet hurt. .. It’s good to eat meat, but not so frequently. And if we look closely at that, for example, cactus is very good for us to be in good health.

## Discussion

Patients and stakeholder groups played an important role in developing recipes following the USDA MyPlate federal dietary and nutrition guidelines, and met patients’ language needs, health literacy levels, and food access challenges. By bringing together patients and key stakeholders invested in addressing social determinants of health (e.g., health educators, community health workers, academics), we created a network of stakeholders with the capacity to disseminate evidence-based nutrition education intended to reduce the impact of chronic conditions among the Latinx and Indigenous Mexican population in rural areas of the desert region of inland southern California. Our work reinforces the value of culturally vetted health education and speak to the need for increased access to evidence-based nutrition education and food products that can be easily incorporated into daily food preparation.

Survey findings indicate that exposure to the cooking demonstrations did not boost diabetes knowledge; however, it did boost participants’ confidence that they could limit their consumption of sugar added to beverages and refined wheat pasta/white rice. This boost in confidence to limit adding sugar to their beverages was statistically significant for indigenous participants but not for Latinx participants and was statistically significant for participants without diabetes but not for those previously diagnosed with diabetes. These results are important since limiting added sugar and refined carbohydrates is consistent with recent dietary recommendations to adopt a more plant-based diet and to limit intake of added sugars [49, 50].

While the diabetes knowledge survey results were not statistically significant, focus group findings indicate that participation in virtual cooking demonstrations helped establish a knowledge base of diabetes or build on existing diabetes knowledge. The cooking demonstrations and the recipes in the cookbook also motivated participants to incorporate more fresh fruits and vegetables into their daily cooking and reduce consumption of added sugar and refined wheat pasta and white rice. Many talked about following recipes in the cookbook and consuming more vegetables after having participated in the virtual cooking demonstration and preparing recipes from the cookbook. Interventions designed to increase study participants’ motivation and self-efficacy to eat more fruits and vegetables have been shown to be effective in increasing fruit and vegetable intake at 14-month follow-up in adolescents [51]. Furthermore, as participants in the focus group indicated, engaging study participants in cooking demonstrations can be particularly effective in teaching community members’ nutrition-related knowledge and skills [52].

As participants’ quotes indicate, vegetables that are part of a pre-Hispanic Mexican diet such as cactus (nopal) [53, 54] motivated participants to eat healthy [55]. Published analyses of cultural heritage and obesity data indicated that the minimally processed, fiber-rich foods characterizing traditional dietary patterns are protective against obesity [49]. People from indigenous communities in Mexico are more likely to retain traditional foodways than Spanish-speaking urban residents of Mexico.

This study had several strengths. Most notable was the use of the ADDIE model to engage patients and key stakeholders in the intervention (i.e., cookbooks with tailored MyPlate-based recipes) designed with major input from the community, with community members featured in the cooking demonstration videos, and the voiceovers in Spanish and Purépecha. Health services researchers are increasingly using this model to incorporate patient and stakeholder perspectives into healthcare interventions [56]. Another strength was a high participant retention rate from pretest to posttest (90%).

Furthermore, survey results indicate participants were conscientious in answering the survey questions. Self-reported height and weight values tend to be unreliable in some populations because of social desirability biases favoring lower weights and greater heights than the values of the same measures collected objectively [57]. Unreliable measures tend to correlate poorly with constructs that theoretically should be highly correlated with them [58]. The fact that diabetes status and hypertension status were significantly or near-significantly correlated with the respondents’ self-reported BMI confirms that the respondents’ self-reported height and weight values were probably accurate. Our subjective impressions were that the questionnaire respondents completed the instrument conscientiously and forthrightly. The survey results support our impression.

Weaknesses of the study include a small, underpowered sample and non-representative recruitment of participants. Additionally, we did not assess years living in the US, which limits understanding of exposure to American diet. Furthermore, this project was carried out during the COVID-19 pandemic. The original study design included in-person meetings (steering council, cookbook workgroup, trainings) and cooking demonstrations in a teaching kitchen. Due to COVID-19 state mandates and university policy, all engagement, dissemination activity, and data collection carried out by the study team had to be conducted virtually. Our community partners, FIND Food Bank, modified their food distribution to a drive-thru approach and the system of provided telehealth and patient check-ins via the phone, both of which limited opportunities to disseminate recipes and nutrition education. The team spent significant time and resources building the capacity of community members to utilize the Zoom conference platform, as well as other virtual platforms, to communicate and disseminate the recipes and nutrition education. While these restrictions placed limitations on the study, stakeholders quickly adapted, and we ultimately adopted a cooking demonstration model used in the community to disseminate the recipes.

Another limitation is the use of only 4 of the 17-items from the MedDiet questionnaire. Initially we intended to administer all 17 questions; however, when we pilot tested the survey participants were critical of how long it took to answer all 17 questions. We therefore decided to limit questions from the MedDiet questionnaire to only those that we felt were most relevant to routine clinical care for patients with pre-diabetes or diabetes.

## Conclusion

This work holds promise for having important public health impact. Our findings indicate that recipes based on MyPlate guidelines tailored to local needs and resources can begin to address the chronic disease burden among rural Latinxm and Indigenous Mexican populations by encouraging increased consumption of vegetables and plant-based proteins. The combined resources of a system of, a community food bank, and academic institutions along with strong community input yielded community-tailored diabetes prevention recipes and community-informed cooking demonstrations of the recipes that were effective in boosting participant self-efficacy to limit weekly consumption of red meat/processed meats. These videos were particularly effective for indigenous participants and for those not already diagnosed with diabetes. While the collaborative approach detailed in this article is labor intensive and requires significant resources and coordination, high-resource and interactive interventions are generally more effective in low-income communities [59]. Our findings indicate this holds true among Latinx and Indigenous Mexican communities where the value of personalism and focus on personal relationships plays a significant role in behavior change around food preparation and daily food intake [60].

## Data Availability

The datasets generated and analyzed during the current study are not publicly available as they are stored on an institutional server but are available from the corresponding author on reasonable request.

## Abbreviations

CBPR: Community Based Participatory Research
